# An Analysis of Stress Concerning Pediatric Emergency Care Nurses

**DOI:** 10.7759/cureus.21299

**Published:** 2022-01-16

**Authors:** Chiaki Toida, Naoto Morimura

**Affiliations:** 1 Department of Disaster Medical Management, The University of Tokyo, Tokyo, JPN

**Keywords:** emergency department operations, nursing skills, pediatric emergencies and critical care, anxiety, patient care

## Abstract

Objective

The aim of this study was an exploratory evaluation of the association between the stressors and stress levels of nurses offering care to critically ill pediatric patients based on their clinical experience and working department in a university hospital.

Methods

The data were collected in October 2018 by administering a self-reporting questionnaire to 169 nurses. The initial analysis compared the anxiety levels between the nurse groups based on their workspace. The next analysis estimated the correlation between the total nursing care and stress levels related to caring for critically ill pediatric patients. We assessed the stress level using the visual analog scale (VAS) score and the total duration of working in the hospital, emergency department (ED), and pediatric department among the three nurse groups.

Results

Overall, 149 (88%) nurses responded to our survey. More nurses from the ED group completed the Advanced Life Support course (19% vs. 3% vs. 7%, p=0.032), and the total VAS scores of the ED group were significantly higher than those of the other groups (median: 80 vs. 56 vs. 54, p=0.005). In the ED group, the total VAS scores negatively correlated with the total duration of working in the hospital (r=-0.292, p=0.022), ED (r=-0.266, p=0.037), and pediatric department (r=-0.505, p<0.001). In the pediatric ward group, the total VAS scores negatively correlated with the total duration of working in the hospital(r=-0.322, p=0.014) and pediatric department (r=-0.375, p=0.004). In the ED group, the proportion of patients who had high anxiety levels with a short duration of working in the pediatric department was significantly higher than that of patients with a long duration of working in the pediatric department (51% vs. 11%, p=0.028).

Conclusions

The ED nurses, especially those with less clinical experience in pediatric care, felt anxious about pediatric emergency care more strongly than those in the other groups, regardless of age and disease. Establishing a pediatric medical care set and conducting off-the-job training might contribute to reducing anxiety related to pediatric emergency care.

## Introduction

Medical practitioners, including nurses, perceive dealing with pediatric patients as difficult and stressful [1,2], especially because children are anatomically and physiologically different from adults owing to the changes they undergo during growth. In addition, the proportion of critically ill pediatric patients aged younger than 18 years comprises a small population compared to the adult patients in Japan [3].

A previous report has shown that nursing is a particularly stressful profession [4]. Furthermore, excessively stressed nurses were less satisfied with their work and ended up providing poorer nursing care quality as a result of this [5]. Therefore, it is essential to appropriately manage the stress levels in the nursing workplace to maintain and improve the quality of nursing care. However, no study in the literature has so far evaluated the differences in perceived stress levels in pediatric nursing care.

The aim of this study was to conduct an exploratory evaluation of the stressors and stress levels of nurses offering care to critically ill pediatric patients according to their clinical experience and working department in a university hospital. The study findings could provide evidence for establishing stress management programs according to the nurse’s background.

## Materials and methods

The study was conducted in October 2018, by administering a self-reporting questionnaire to all the 169 nurses across the three departments, including 74 nurses in the emergency department (ED), 27 nurses in the dedicated emergency intensive care unit (ICU) for pediatric patients, and 68 nurses in the pediatric wards.

The three-part questionnaire was designed to evaluate the stress levels of nurses offering care to critically ill pediatric patients, and the system that nurses need to establish to reduce their anxiety based on their clinical experience. Part 1 inquired about their clinical experience, as follows: length of working in the hospital/ED/pediatric department and their completion of off-the-job training courses, including Basic Life Support (BLS)/Advanced Life Support (ALS)/Pediatric Advanced Life Support (PALS)/Japan Nursing for Trauma Evaluation and Care (JNTEC). Part 2 comprised nine questions about the stress levels of nurses offering care to critically ill pediatric patients. We assessed the stress levels using the visual analog scale (VAS) [6,7], which involves a straight horizontal line of a fixed length of 10 cm. The ends are defined as the extreme limits of the parameter to be measured; 0 cm implies no anxiety about pediatric care and 10 cm implies maximum anxiety (Appendix 1). The highest possible VAS score was 90 points for all the nine questions, which were rated on a scale of 0−10 points each. Part 3 inquired about the potential clinical system that needs to be established to resolve the anxiety related to pediatric emergency care.

The first analysis compared the clinical experiences, the anxiety level, and clinical system between the nurse groups based on their workspace, particularly the ED, emergency ICU for pediatric patients, and the pediatric ward. The nurses were divided into three groups based on their workspace during the survey period. The next analysis estimated (1) the correlation between the total VAS score and the total duration of working in the hospital; (2) the correlation between the total VAS score and the total duration of working in the ED; (3) and the correlation between the total VAS score and the total duration of working in the pediatric department among the three nurse groups.

Continuous variables are presented as median with interquartile ranges (25th-75th percentile), and categorical variables as numbers and percentages of patients. The Kruskal-Wallis test with multiple comparisons was used for continuous variables, whereas Fisher's exact test with multiple comparisons was used for categorical variables. Correlations between variables were analyzed using Spearman’s rank correlation. Because total VAS scores and years of experience would not be normally distributed, we dichotomized these variables by each median value and used Fisher’s exact test to evaluate the association between the level of anxiety and clinical experience. All statistical analyses were performed using STATA/SE software, version 16.1 (StataCorp, College Station, TX). A two-tailed p-value <0.05 indicated statistical significance.

The protocol for this research project was approved by the Ethics Committee of The University of Tokyo and conforms to the provisions of the Declaration of Helsinki. Approval No: 2019262NI.

## Results

Overall, 149 (88%) nurses responded to our survey within one month without any reminders. The lengths of working in the hospital, ED, and pediatric department were six years (3-12), zero years (0-4), and one year (0-5), respectively. The proportion of nurses who completed the BLS, ALS, PALS, and/or JNTEC courses were 36%, 11%, 11%, and/or 1%, respectively. The total VAS score, which assessed anxiety about pediatric emergency care, was 63 points (46-80). The following proportion of nurses required the various clinical systems: off-the-job training for critically ill pediatric patients: 89%; pediatric medical care sheet for choosing an adequate dosage of medicine and size of equipment per patients’ physique: 73%; pediatric medical care set of equipment according to pediatric patients’ physique: 55%; network for smooth cooperation with the relevant department: 60%; and clinical conference for critically pediatric patients: 36%. Comparison between the three nurse groups is presented in Tables 1, 2.

**Table 1:** Comparison of the clinical experiences, anxiety level, and clinical systems between the nurse groups ^*†^There is a significant difference among the three groups based on ANOVA/Bonferroni test

Variables	Total	Emergency department group	Emergency ICU group	Pediatric ward group	P-value
	(n=149)	(n=62)	(n=29)	(n=58)	
Duration in years, median (interquartile range)					
Working in the hospital	6 (3-13)	9 (5-14)	4 (2-6)	6 (3-13)	0.587
Working in the emergency department	0 (0-4)	4 (1-6)*†	0 (0-0)*	0 (0-0)†	0
The total length of working in the pediatric department	1 (0-5)	0 (0-0)*†	3 (1-5)*	4.5 (2-8)†	0.002
Number of nurses who completed training courses, n (%)					
Basic Life Support	53 (36)	21 (34)	8 (28)	24 (41)	0.419
Advanced Life Support	17 (11)	12 (19)*†	1 (3)*	4 (7)†	0.032
Pediatric Advanced Life Support	17 (11)	7 (11)	8 (28)	2 (3)	0.248
Japan Nursing for Trauma Evaluation and Care	2 (1)	1 (2)	1 (3)	0	0.408
Number of nurses who required the various clinical systems, n (%)					
Off-the-job training for critically ill pediatric patients	132 (89)	54 (87)	25 (86)	53 (91)	0.689
Pediatric medical care sheet for choosing an adequate dosage of medicine and size of equipment per patients' physique	109 (73)	44 (71)	22 (76)	43 (74)	0.866
Pediatric medical care set of equipment according to pediatric patients' physique	82 (55)	38 (61)	11 (38)	33 (57)	0.106
Network for smooth cooperation with the relevant department	89 (60)	33 (53)	21 (72)	35 (60)	0.219
Clinical conference for critically pediatric patients	54 (36)	24 (39)	6 (21)	24 (42)	0.145

**Table 2:** Comparison of the anxiety levels about pediatric emergency care between the nurse groups ^*†^There is a significant difference among the three groups based on ANOVA/Bonferroni test

Variables	Total	Emergency department group	Emergency ICU group	Pediatric ward group	P-value
	(n=149)	(n=62)	(n=29)	(n=58)	
Total visual analog scale score, point, median (interquartile range)	63 (46-80)	80 (65-90)*†	56 (45-63)*	54 (40-67)†	0.005
Q1. Anxiety about evaluating normal vital signs for pediatric patients, point, median (interquartile range)	6 (3-8)	8 (6-10)	5 (3-6)	4 (3-6)	0.058
Q2. Anxiety about choosing the appropriate size of a medical device for pediatric patients, point, median (interquartile range)	6 (5-9)	8 (7-10)	5 (4-6)	5 (3-7)	0.369
Q3. Anxiety about choosing the appropriate medicine dose for pediatric patients, point, median (interquartile range)	8 (7-10)	10 (8-10)	7 (6-8)	8 (6-9)	0.324
Q4. Anxiety about treating pediatric patients younger than one year, point, median (interquartile range)	7 (4-10)	10 (7-10)*†	5 (4-7)*	5.5 (3-8)†	0.048
Q5. Anxiety about treating pediatric patients aged between one to six years, point, median (interquartile range)	6 (4-9)	9 (7-10)*†	5 (4-6)*	5 (3-7)†	0.039
Q6. Anxiety about treating pediatric patients aged older than six years, point, median (interquartile range)	5 (4-8)	8 (6-10)*†	5 (4-5)*	5 (3-6)†	0.014
Q7. Anxiety about treating pediatric patients with a critical endogenous disease, point, median (interquartile range)	8 (5-10)	10 (8-10)*†	6 (6-8)*	5.5 (4-8)†	0.011
Q8. Anxiety about treating pediatric patients with a critical exogenous disease, point, median (interquartile range)	8 (6-10)	9.5 (7-10)	6 (5-8)	8 (6-10)	0.464
Q9. Anxiety about treating pediatric patients with cardiopulmonary arrest, point, median (interquartile range)	10 (8-10)	10 (8-10)	9 (8-10)	9.5 (8-10)	0.189

**Figure 1:**
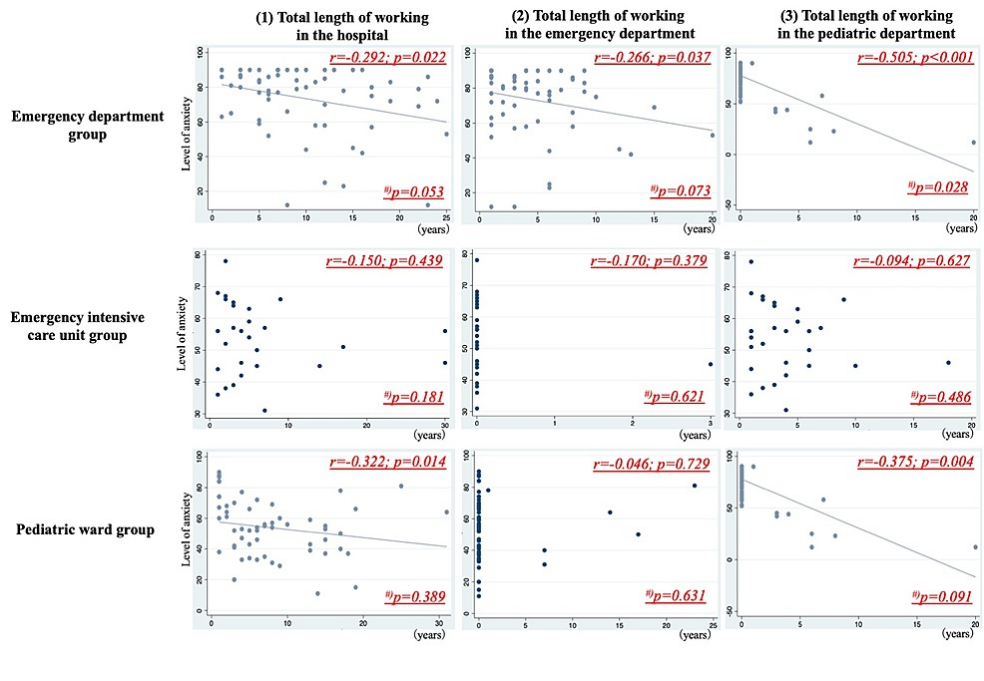
Correlation between the total length of working and the anxiety level In the ED group, the VAS scores negatively correlated with the total length of working in the hospital, ED, and the pediatric department (r=-0.292; p=0.022, r=-0.266; p=0.037, r=-0.255; p<0.001). In the pediatric ward group, the total VAS scores negatively correlated with the total length of working in the hospital and pediatric department (r=-0.322; p=0.014, r=-0.375; p=0.004) #) shows the p-value of Fisher’s exact test. According to Fisher’s exact test, in the ED group, the proportion of patients who had high anxiety levels with a short length of working in the pediatric department was significantly higher than that of patients with a long length of working in the pediatric department (51% vs. 11%, p=0.028)

Compared to the other groups, the ED group had the highest proportion of nurses who completed the ALS course (ED group vs. emergency ICU group vs. pediatric ward group: 19% vs. 3% vs. 7%, p=0.032), and the total VAS score of the ED group was high [80 points (65-90) vs. 56 points (46-63) vs. 54 points (40-67), p=0.005]. The ED group nurses felt more anxious about treating critically ill pediatric patients, regardless of age. Moreover, the anxiety levels about treating critically ill pediatric patients with endogenous diseases were significantly different among the groups. However, the anxiety levels about treating pediatric patients with exogenous diseases and cardiopulmonary arrest were not significantly different among the groups; thus, even nurses in the pediatric ward who treat pediatric patients regularly had high anxiety levels related to treating those patients with low frequency. The association between the level of anxiety and clinical experience is shown in Figure 1.

## Discussion

This study showed that the ED group felt anxious about pediatric emergency care, regardless of age and disease, more strongly than the other nurse groups. Moreover, there were negative correlations between the level of anxiety and clinical experiences, particularly in the pediatric department. Our findings also showed that even nurses in the pediatric ward who treat pediatric patients regularly have high anxiety levels about treating pediatric patients with exogenous diseases and cardiac arrest if the experience is low. These findings suggest that off-the-job training may be needed to compensate for the lack of experience in the actual clinical setting.

Unlike adults, children have unique standards for drug dosing and medical equipment size, based on their anthropometrics. Previous studies have demonstrated that medication-dosing errors occurred more frequently for pediatric than adult patients, especially in the emergency setting [8-10]. Moreover, medical staff with less experience in pediatric care committed medical errors among critically pediatric patients more frequently than those with more experience [10]. Since critically ill pediatric patients are few [1], the medical staff has received insufficient clinical experience in pediatric emergency care, which makes them perceive pediatric critical care as difficult [3]. In this study, ED nurses with a shorter duration of working, particularly in the pediatric department, were more anxious about pediatric emergency care. Even nurses who regularly worked in the pediatric ward felt anxious about treating critically ill pediatric patients with exogenous diseases.

Previous studies have reported that excessively stressed nurses provide poor quality of care. Therefore, it is essential to appropriately manage their stress by reducing the workload by improving clinical systems, strengthening work relationships, and imparting nursing skills and knowledge [5,11]. Firstly, a medical care set can be developed to help select the optimal medication dosage and equipment size for critically ill pediatric patients according to their physique. Several reports have shown that the pediatric medical care set, based on their body size, reduced medication errors and improved the quality of pediatric emergency care [12-14]. Based on this, we developed a pediatric medical care set, including medical care sheets commonly used in all departments.

A previous report has suggested that developing a pediatric medical care set reduced anxiety about selecting the adequate medication dosage and equipment size based on the body size. However, this did not reduce the anxiety about treating pediatric patients in actual clinical practice [3]. In this study, most nurses (89%) were required to undergo off-the-job training for critical pediatric care to reduce anxiety about pediatric emergency care (Table 1). Education and training are essential for consistently providing high-quality medical care. However, the rarity of critically ill pediatric patients makes it difficult to maintain and practice the necessary know-how [1,11]. To address this, off-the-job training was recommended to facilitate knowledge retention and reduce anxiety about pediatric emergency care. Moreover, a previous study has reported that during off-the-job training, involving the entire staff in the decision-making and planning was beneficial for establishing a clinical network with effective communication, resulting in high-quality pediatric emergency care.

This study has some limitations. Firstly, the generalizability of the findings might be limited due to its single-center design. Secondly, this survey was limited to findings from the questionnaires. Thus, the nurses’ anxiety about pediatric emergency care in the clinical setting was not evaluated. Further studies are required to identify specific stressors and help devise methods to manage stress and anxiety in pediatric emergency care.

## Conclusions

This study suggests that the ED nurse group felt anxious about pediatric emergency care, regardless of age and disease, more strongly than the other nurse groups. Moreover, there were negative correlations between the level of anxiety and clinical experience, particularly in the pediatric department. The anxiety about pediatric emergency care may be reduced by requiring nurses to accomplish more than half of the pediatric medical care sheet and set, providing off-the-job training where the entire staff is involved in decision-making and planning, and establishing effective communication to provide high-quality pediatric emergency care.

## Appendices

Appendix 1. The questionnaire form

List of question items

Part 1. Questions about the clinical experience

Q1. Length of working in the hospital/emergency department/pediatric department

Q2. Completion of the off-the-job training course including Basic Life Support/Advanced Life support/Pediatric Advanced Life Support/Japan Trauma Evaluation and Care

Part 2. Questions about the stress levels of nurses offering care to critically ill pediatric patients based on the visual analog scale scores 

Q1. Anxiety about evaluating normal vital signs for the pediatric patient

Q2. Anxiety about choosing the appropriate size of a medical device for the pediatric patient

Q3. Anxiety about choosing the appropriate medicine dose for the pediatric patient

Q4. Anxiety about treating pediatric patients younger than one year in age

Q5. Anxiety about treating pediatric patients aged between one to six years

Q6. Anxiety about treating pediatric patients aged more than six years

Q7. Anxiety about treating pediatric patients with a critical endogenous disease

Q8. Anxiety about treating pediatric patients with a critical exogenous disease

Q9. Anxiety about treating pediatric patients with cardiopulmonary arrest

Part 3. Questions about the clinical system which you want to establish for dissolving anxiety for pediatric emergency care
